# Iberian-Appalachian connection is the missing link between Gondwana and Laurasia that confirms a Wegenerian Pangaea configuration

**DOI:** 10.1038/s41598-020-59461-x

**Published:** 2020-02-12

**Authors:** Pedro Correia, J. Brendan Murphy

**Affiliations:** 1 Institute of Earth Sciences, Pole of the Faculty of Sciences, University of the Porto, Porto, Portugal; 2 Department of Earth Sciences, St. Francis Xavier University, Antigonish, Nova Scotia Canada

**Keywords:** Palaeoclimate, Geodynamics, Geomorphology, Palaeontology, Tectonics

## Abstract

The formation and subsequent breakup of the supercontinent Pangaea has dominated Earth’s evolution for the last 320 million years. Although its configuration at the time of breakup is widely accepted, there remains uncertainty about its configuration at the time of its amalgamation. The classic Pangaea-A model, widely known as “Wegenerian” configuration, implies that Pangaea did not deform internally between amalgamation and breakup. Palaeomagnetic studies suggest the possibility of a Pangaea-B configuration, in which Gondwana was located about 3000 km farther east relative to Laurasia compared its location in Pangaea-A. Here, we provide firm evidence of an Iberian-Appalachian connection in the Late Pennsylvanian (307–299 Ma) which confirms a Pangaea-A configuration for the relative locations of Gondwana and Laurasia in the late Palaeozoic, negating the possibility of Pangaea-B at that time. This evidence is based on palaeobotanical and biostratigraphic findings recently documented in the Carboniferous successions of Iberia (Douro Basin, Portugal). These new findings also precisely constrain the timing of uplift of the Appalachian and Iberian (Variscan) orogens and climatic changes during the amalgamation of Pangaea and final closure of the Rheic Ocean.

## Introduction

Over the past 30 years, a broad consensus has emerged that repeated cycles of supercontinent amalgamation and dispersal have occurred since the end of the Archean, and these cycles have profoundly affected the Earth’s evolution^1,2^. Less clear is whether the supercontinent changes its configuration during its existence due to internal stresses.

**Figure 1:**
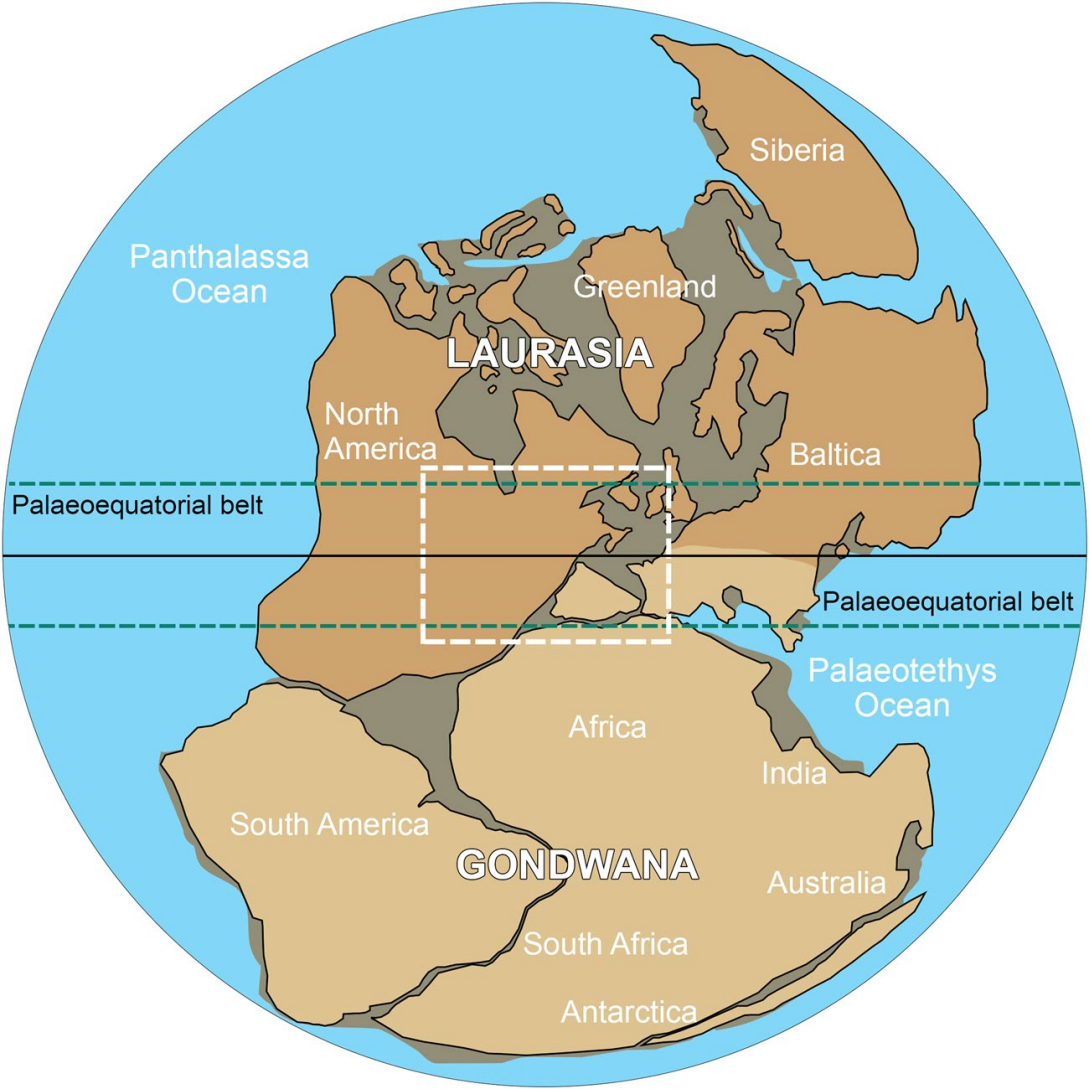
Idealized Pangaea-A (“Wegenerian”) configuration based on continental connection between eastern Laurentia (Laurasia) and Iberia (northwestern Gondwana^20–33^) in the late Palaeozoic (adapted from^9^). Colour legend for the image: blue: Oceans; light brown: Gondwana; dark brown: Laurasia; grey: shallow seas and coastal/flooded areas.

**Figure 2:**
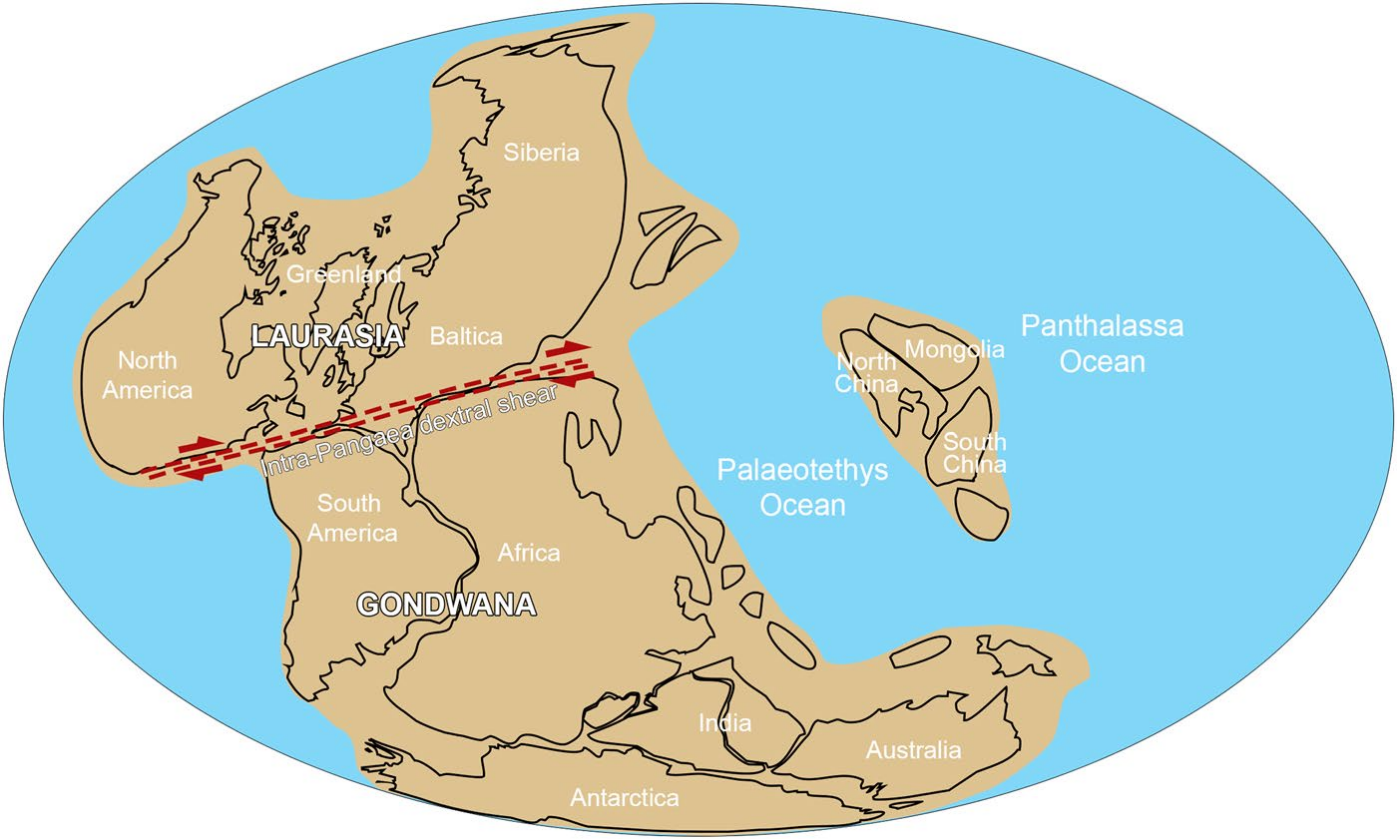
Late Palaeozoic Pangaea-B configuration in which Gondwana is located about 3000 km farther east relative to Laurasia (adapted from^9^).

Although the classical “Wegenerian” configuration of Pangaea immediately prior to its Early Mesozoic breakup is well constrained, there remains uncertainty about its late Palaeozoic configuration. Two end member models have emerged; Pangaea-A, which is essentially the “Wegenerian” fit (A-1^3^; A-2^4^), and Pangaea-B, based on palaeomagnetic data^5^, in which Gondwana was located about 3000 km farther east relative to Laurasia, compared to the Pangaea-A configuration (Fig. 1). A late Palaeozoic Pangaea-B configuration (Fig. 2) would require substantial lateral (dextral) shear along major faults, inferred by Irving^5^ to have occurred between the middle Carboniferous and Late Triassic, in order to obtain the Wegenerian configuration before Pangaea breakup. More recent palaeomagnetic data have been used to support the transition from a Pangaea-B to a Pangaea-A configuration during the Permian^6,7^, and in the most recent model^8^, the transition occurred between 275 and 260 Ma. However, geologic evidence that would distinguish between these hypotheses is lacking. Moreover, the validity of the palaeomagnetic data purported to support the Pangaea-B configuration has recently been challenged^9^.

The collision between Laurasia and Gondwana during the Late Devonian-early Permian was a key event in the amalgamation of Pangaea and resulted in the destruction of the Rheic Ocean and the formation of the Appalachian and Variscan (Hercynian) orogens in the interior of Pangaea^10,11^. A key element in reconstructing palaeogeographic environments is to examine the first appearance of shared flora between continents. For example, the occurrence of the Permian flora *Glossopteris* has been crucial in understanding the configuration of Gondwana^12,13^. The confinement of this flora to Gondwana and its absence from Laurasia has been attributed to the presence of physical barriers (e.g. distance, mountain ranges, climate/latitude) that may have restricted its migration^13^.

However, determination of the palaeogeography of Laurasia relative to Gondwana during the late Palaeozoic is hindered by the lack of palaeobiogeographic evidence linking both continents. In this paper, we draw on recent discoveries in Carboniferous successions in the Iberian Massif (Douro Basin, Portugal)^14,15^ that, for the first time, provide linkages between the ancient landmasses Laurentia and Iberia (located along the northern margin of Gondwana) along the palaeoequatorial belt during the Late Pennsylvanian (307–299 Ma) (Fig. 1). In so doing, we provide palaeobotanical and biostratigraphic evidence that the Pangaea-A configuration was in place at that time, negating the possibility of Pangaea-B configuration in the late Palaeozoic.

## Iberian-Gondwanan Connection

Models for Variscan orogenesis and Pangaea amalgamation rely on ca. 420–320 Ma continental reconstructions. At ca. 420 Ma, reconstructions primarily influenced by palaeomagnetic data^16,17^ show Gondwanan terranes, including Iberia rifted from the northern Gondwanan margin thereby forming the Palaeotethys Ocean^18,19^. Other reconstructions, however, based on a wealth of faunal, lithological, stratigraphic, detrital zircon and palaeoclimatic data^20–25^, imply that these terranes remained along the Gondwanan margin for the entirety of the Palaeozoic^26–29^. In the latter scenario, Rheic Ocean closure resulted from continental collision of Laurasia with the northern Gondwanan margin, which began ca. 380 Ma^25,30^. Iberia preserves a continuous Early Ordovician to Late Devonian passive margin sequence^31,32^ including typically Gondwanan Late Ordovician glaciomarine deposits^33^, and lacks ca. 420 Ma rift-drift deposits predicted by the formation of the Palaeotethys Ocean. On the basis of this evidence, we adopt the second scenario and our reconstructions showing a unified Iberia and Gondwana throughout the Palaeozoic.

## Palaeofloral and Biostratigraphic Evidence

**Figure 3:**
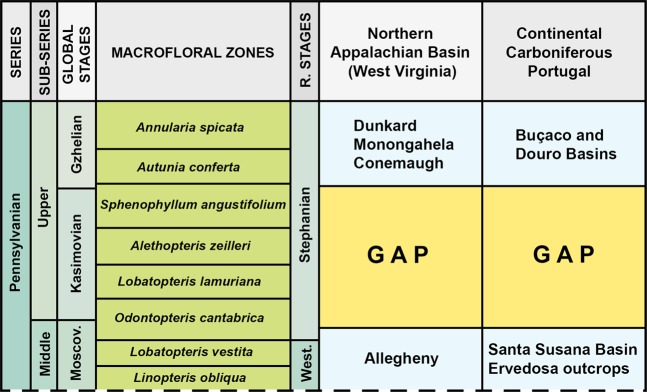
Biostratigraphic constraints between Laurentia and Iberia including a macrofloral biostratigraphic gap correlated between the Upper Pennsylvanian successions of Appalachian region in West Virginia and Iberia in Portugal. Diagram modified from^15^. Abbreviations: R.: regional; Moscov.: Moscovian; West.: Westphalian.

Abundant Carboniferous-Permian floras and palaeoenvironmental/climatic distribution data have been identified in Laurasia^13,15,34–36^. Detailed studies^15,34,37^ of flora that demonstrate significant affinities between the Pennsylvanian (late Moscovian and Gzhelian) floras of North America and Iberian Massif are interpreted to reflect a proximal palaeobiogeography between Laurentia and Iberia within the palaeoequatorial belt. Biostratigraphic studies^34,38^ identify the existence of a macrofloral biostratigraphic gap for the Kasimovian stage in the Appalachian region in West Virginia Basin (USA) correlated with the Upper Pennsylvanian of Portugal^15^ (Fig. 3). This gap is documented in parts of the palaeoequatorial belt during the Kasimovian and is attributed to a lowstand reflecting a major glaciation event in southern Gondwana^15,34^.

**Figure 4:**
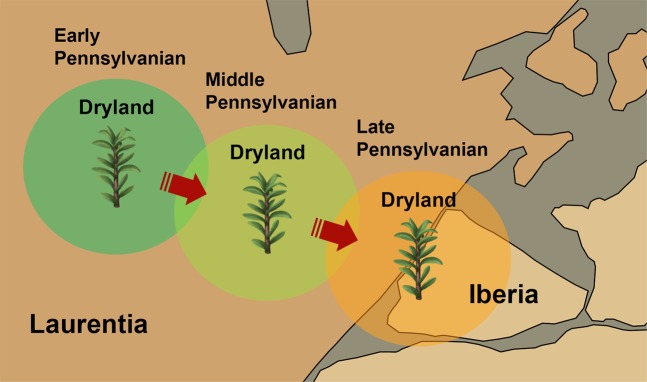
Palaeoenvironmental and palaeoclimatic constraints and floral migration between Laurentia and Iberia within Pangaea-A. Enlarged view of central Pangaea in Fig. 1 (white rectangular box area) showing the emergence of “dryland” environments at varying spatial and temporal scales and diachronous migration of dry-climate adapted flora like *Lesleya* between the Laurentian and Iberian landmasses. *Lesleya*-fossil record data for the floral migration route are from^14,40–43,51,52^.

Carboniferous-Permian floras, restricted to same type of palaeoenvironments shared by Laurentia and Iberia, are key elements to determine the palaeogeography of Pangaea as it amalgamated. Determination of land bridges linking Laurentia and Iberia for floral exchange attests to the importance of constraining the palaeoenvironmental and palaeoclimatic conditions between these continental lands in the interior of Pangaea. Such constraints are provided by the floras that were restricted to “dryland” environments located in the tropical regions of central Pangaea and lived in both Laurentia and Iberia. Cycadopsid *Lesleya*, a rare Carboniferous-early Permian seed-plant of the Euramerican realm, was a dry-climate adapted flora (known as “dryland flora”) restricted to tropical dryland environments of central Pangaea^14,39–43^ (Fig. 4).

Pangaean tropical regions experienced major cyclic environmental changes during the Pennsylvanian-early Permian interval, with significant modifications to ecosystems and biotic communities (biotic stress) resulting from alternation of wetland and dryland floras. Such changes were a result of glacial and interglacial cycles, and their effects were especially felt in the tropical regions of central Pangaea during this interval^35,40,41,43–49^. The dryland environments occupied part of the tropical landscapes of central Pangaea during the Pennsylvanian^14,35,40–43^ (Fig. 4). The emergence of these environments is intricately linked to a warmer or drier climate during interglacial periods^46,49,50^. These interglacial periods led to significant changes in climate and therefore the overall composition of resident floral assemblages in the tropical regions of central Pangaea in the late Palaeozoic^34,35,40,41,43,47,49^.

Fossils of *Lesleya* have been widely documented in Early-Middle Pennsylvanian-age dryland basins of North America^14,40–43,51,52^. Recent discoveries in the Upper Pennsylvanian of Portugal have documented the first occurrence of *Lesleya* in Iberian Massif^14^. The Portuguese *Lesleya* specimens were found in lower Gzhelian strata of the Douro Basin and occur in intramontane deposits that preserve evidence of dry climate^14^. Dry climate is characterized by the moisture-deficient (dryness) and well-drained conditions^14,15^. The appearance of *Lesleya* in Iberia (Fig. 4) coincided with the onset of an interglacial interval in the Kasimovian-Gzhelian (304 Ma) after the waning of a major glaciation in southern Gondwana^35,40,53,54^. As a result, parts of palaeoequatorial belt especially of central Pangaea, where eastern Laurentia and Iberia were located, became drier and less humid during the Gzhelian (Late Pennsylvanian, 304–299 Ma)^34,35,55^ (Fig. 4).

Other typical dryland floras such as the walchian conifers *Walchia* and *Ernestiodendron*, cordaitalean *Cordaites*, callipterid peltasperms *Autunia conferta* and *Rhachiphyllum*, and the dicranophyllalean *Dicranophyllum* also flourished at various places in Laurentia (e.g. West Virginia) and Iberia. Such dryland biomes were more abundant during periods of warm or dry climate in the Late Pennsylvanian and early Permian^14,15,34,37,38,40,49,50,56^. These palaeobotanical data provide palaeogeographic constraints on the proximity of Laurentia and Iberia and are key to distinguishing between the competing Pangaea configurations.

## The ‘Missing link’: Resolving Pangaea-A versus Pangaea-B Controversy

**Figure 5:**
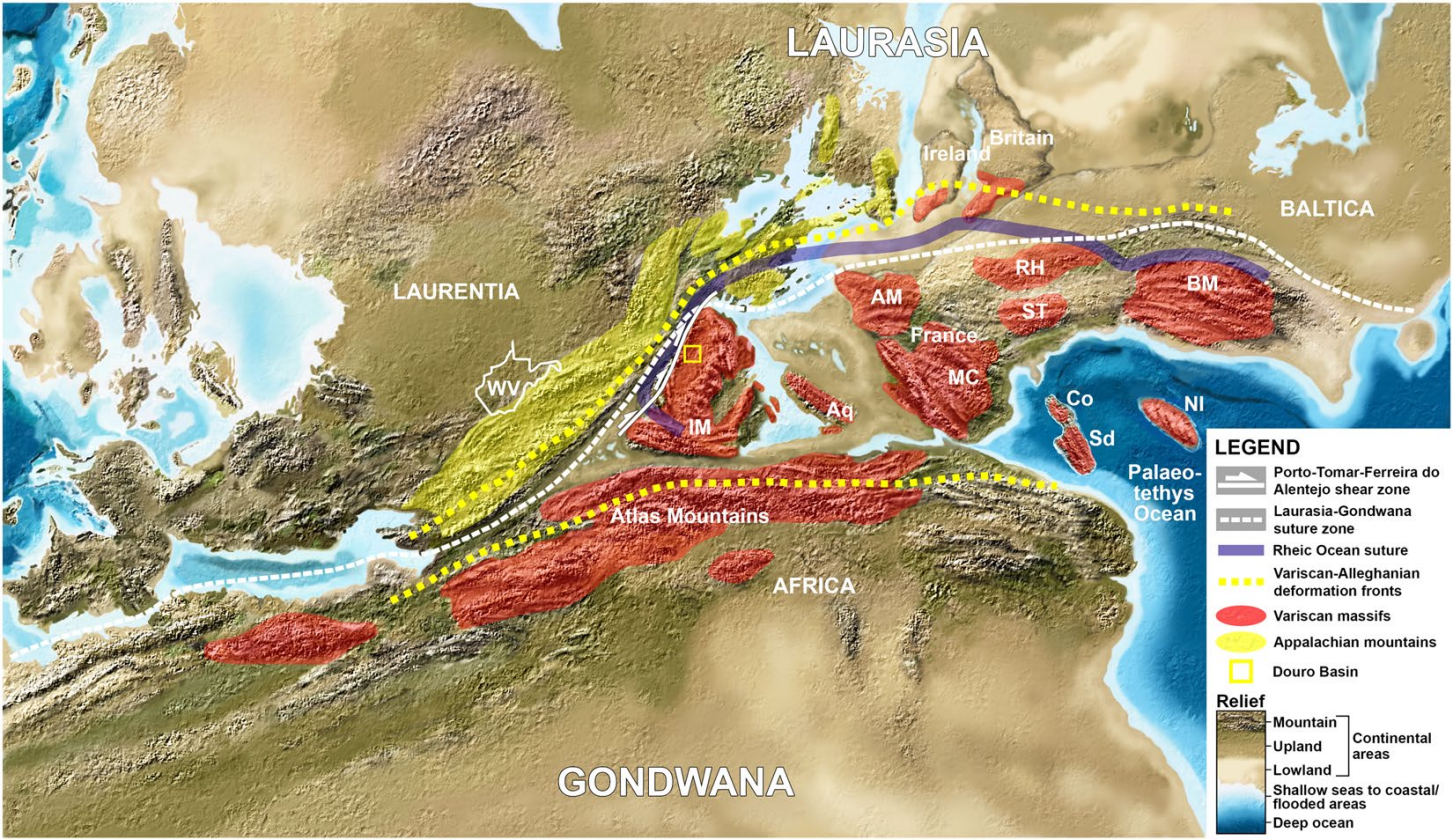
Palaeogeographic and palaeotopographic constraints within Pangaea-A showing the continental linkage between eastern Laurentia and Iberia and uplift of the Appalachian and Variscan orogens in the late Gzhelian-early Permian. Topography adapted from North American 300 MA © 2013 Colorado Plateau Geosystems Inc (https://deeptimemaps.com/). Data for the legend are from^57–61^. Abbreviations: WV: West Virginia; IM: Iberian Massif; Aq: Aquitaine; AM: Armorican Massif; MC: French Central Massif; RH: Rheno-Hercynian terrane; ST: Saxo-Thuringian terrane; BM: Bohemian Massif; Sd: Sardinia (Italian island); Co: Corsica (French Mediterranean island); NI: Variscan basement of northern Italy.

The Pangaea-A versus Pangaea-B controversy underscores large uncertainties about the palaeogeographic position of Gondwana relative to Laurasia in the Late Devonian-early Permian interval. Recent palaeobotanical and biostratigraphic studies^14,15^ indicate a proximal Iberian-Appalachian palaeogeography in the Late Pennsylvanian. Such evidence provides significant constraints in the palaeogeography, palaeoclimate and palaeotopography in both the Appalachian and Iberian (Variscan) orogens (Fig. 5).

Because they are indicators for climatic and environmental conditions^14,39–43^, the occurrence of dryland floras typical from North America such as *Lesleya* in the Upper Pennsylvanian strata of Portugal is evidence of migration of dry-climate adapted floras between the Laurasian and Gondwanan continents. This floral migration suggests that eastern Laurentia and Iberia were connected or geographically very close, sharing the same tropical dryland environment within central Pangaea in the Late Pennsylvanian (Fig. 4). Moreover, the appearance of *Lesleya* in the early Gzhelian (Late Pennsylvanian, 304–301 Ma) of Iberia, immediately after a transition from glacial to interglacial conditions in the Kasimovian-Gzhelian interval (304 Ma)^40,53,54^, indicates that this flora migrated from Laurentia to Iberia, possibly when new dryland habitats appeared (Fig. 4). In this proximal configuration, Iberia probably acted as a migratory option or refuge to the many dry-climate adapted floras of Laurentia, perhaps because conditions of greater dryness had prevailed in Iberia in the early Gzhelian^14^. During that time interval, new dryland species such as *Lesleya iberiensis* emerged in the Iberia in well-drained, moisture-deficient environments^14^.

The migration routes of dryland flora between Laurentia and Iberia (Fig. 4) provide insights into the location and timing of uplift of the Appalachian and Variscan orogens during continental collision between Laurasia and Gondwana during the amalgamation of Pangaea (Figs. 1 and 5). These migration routes were influenced by climate and tectonically-induced topographic changes. As mountain ranges acted as physical barriers to the floral exchanges^13^ between Laurentia and Iberia within central Pangaea, this migration occurred before uplift of the Appalachian and Variscan orogens, i.e. during the early Gzhelian (Late Pennsylvanian, 304–301 Ma) (Fig. 4). This palaeobiogeographic connection records early stages of uplift during the assembly and amalgamation of Pangaea and implies a connection along the palaeoequatorial belt between the Appalachian orogen and the Variscan orogen in Iberia (Fig. 5). A macrofloral biostratigraphic gap correlated between the Upper Pennsylvanian successions of Appalachian region in West Virginia and Portugal^15^ (Fig. 3) supports an Iberian-Appalachian connection at that time. The timing of this connection implies that uplift of the Appalachian and Variscan orogens occurred during the late Gzhelian (Late Pennsylvanian) to Asselian (early Permian) (301–295 Ma).

Our data provide the ‘missing link’ between Gondwana and Laurasia during the final amalgamation of the supercontinent Pangaea in the Late Pennsylvanian and confirms a Pangaea-A (“Wegenerian”) configuration at that time (Fig. 1). Consequently, these results indicate that the palaeomagnetic data used to support a Pangaea-B configuration (Fig. 2) in the late Palaezoic^5^ represent an artifact of data quality, geometrical fits used to restore the Atlantic-bordering continents to one another, and processes such as inclination shallowing in clastic rocks, as suggested by Domeier *et al*.^9^.
